# Career choice and influential factors among medical students majoring in psychiatry in China

**DOI:** 10.1186/s12909-021-02622-x

**Published:** 2021-03-25

**Authors:** Ying-Jian Zhang, Kai Yuan, Su-Hua Chang, Wei Yan, Jian-Yu Que, Jia-Hui Deng, Yi-Miao Gong, Jia-Ming Luo, Shi-Chang Yang, Cui-Xia An, Yi-Min Kang, Hua-Shan Xu, Yi-Ming Wang, Li-Fang Zhang, Wen-Fang Zhang, Yin-Li Song, Dong-Wu Xu, Huan-Zhong Liu, Wen-Qiang Wang, Chuan-Xin Liu, Wen-Qiong Yang, Liang Zhou, Jiu-Bo Zhao, Miao-Yu Yu, Jun-Yu Chen, Hong Tang, Juan Peng, Xiu-Jun Zhang, Yong Xu, Ning Zhang, Li Kuang, Zhan-Jiang Li, Yu-Hua Wang, Jie Shi, Mao-Sheng Ran, Yan-Ping Bao, Le Shi, Lin Lu

**Affiliations:** 1 Peking University Sixth Hospital, Peking University Institute of Mental Health, NHC Key Laboratory of Mental Health (Peking University), National Clinical Research Center for Mental Disorders (Peking University Sixth Hospital), 51 Huayuan Bei Road, Beijing, 100191 China; 2 Peking-Tsinghua Center for Life Sciences and PKU-IDG, McGovern Institute for Brain Research, Beijing, China; 3 School of Psychiatry, North Sichuan Medical College, Nanchong, China; 4 Department of Psychiatry, Second Affiliated Hospital of Xinxiang Medical University, Xinxiang, China; 5 Department of Psychiatry, First Hospital of Hebei Medical University, Mental Health Institute of Hebei Medical University, Brain Ageing and Cognitive Neuroscience Laboratory, Hebei Medical University, Shijiazhuang, China; 6 School of Basic Medical Sciences, Inner Mongolia Medical University, Hohhot, China; 7 School of Mental Health, Bengbu Medical College, Anhui, China; 8 Department of Psychiatry, Affiliated Hospital of Guizhou Medical University, Guiyang, Guizhou China; 9 Department of Neurology, Changzhi People’s Affiliated Hospital of Changzhi Medical College, Changzhi, Shanxi China; 10 Mental Health Department of Baotou Medical College, Inner Mongolia University of Science and Technology, Baotou, China; 11 Department of Pathology, Daqing Campus of Harbin Medical University, Daqing, China; 12 School of Mental Health, Wenzhou Medical University, Wenzhou, Zhejiang China; 13 Chaohu Hospital of Anhui Medical University, Anhui, China; 14 Xianyue Hospital of Xiamen, Xiamen, Fujian China; 15 Jining Medical University, Jining, China; 16 Department of Neurology, Dongfeng Hospital, Hubei University of Medicine, Shiyan, Hubei China; 17 Affiliated Brain Hospital of Guangzhou Medical University (Guangzhou Huiai Hospital), Guangzhou, China; 18 Department of Psychology, School of Public Health, Southern Medical University, Guangzhou, China; 19 Department of Psychiatry, Zhujiang Hospital, Southern Medical University, Guangzhou, China; 20 Department of Mental Health, Second Affiliated Hospital of Guangxi Medical University, Nanning, Guangxi China; 21 Shenzhi Department, Fourth Affiliated Hospital of Xinjiang Medical University, Urumqi, China; 22 Department of Psychology, Gannan Medical University, Ganzhou, Jiangxi China; 23 Teaching and Research Section of Psychology, School of Management, Zunyi Medical University, Zunyi, Guizhou China; 24 North China University of Science and Technology Tangshan, Hebei, China; 25 Shanxi Key Laboratory of Artificial Intelligence Assisted Diagnosis and Treatment for Mental Disorders, First Hospital of Shanxi Medical University, Taiyuan, China; 26 Department of Psychiatry, First Hospital/First Clinical Medical College of Shanxi Medical University, Taiyuan, China; 27 Brain Hospital of Nanjing Medical University, Nanjing, Jiangsu China; 28 Mental Health Center, University-Town Hospital of Chongqing Medical University, Chongqing, China; 29 Department of Psychiatry, First Affiliated Hospital of Chongqing Medical University, Chongqing, China; 30 Department of Clinical Psychology, National Clinical Research Center for Mental Disorders, Beijing, China; 31 Beijing Key Laboratory of Mental Disorders, Beijing Anding Hospital, Capital Medical University, Beijing, China; 32 Center of Schizophrenia, Beijing Institute for Brain Disorders, Beijing, China; 33 Advanced Innovation Center for Human Brain Protection, Capital Medical University, Beijing, China; 34 Department of Psychiatry, Qiqihar Medical University, Qiqihar, China; 35 National Institute on Drug Dependence, Peking University, Beijing, China; 36 Department of Social Work and Social Administration, University of Hong Kong, Hong Kong, China; 37 National Institute on Drug Dependence, School of Public Health, Peking University, 38 Xueyuan Road, Beijing, 100191 China

**Keywords:** Career choice, Medical students, Psychiatry major, Medical education, Specialty choice

## Abstract

**Background:**

The undergraduate program of psychiatry has been widely established in recent years to improve the education and recruitment of psychiatrists in China. We aim to investigate the career choice of medical students majoring in psychiatry in China and the influential factors.

**Method:**

This multicenter study was conducted in 26 medical schools in China from May to October of 2019. Participants included 4610 medical students majoring in psychiatry and 3857 medical students majoring in clinical medicine. Multivariable logistic regression was used to investigate the influential factors of students’ choices of psychiatry at matriculation and as a career.

**Results:**

44.08% of psychiatry majored students gave psychiatry as a first choice at matriculation, and 56.67% of them would choose psychiatry as a career, which was in sharp contrast to the proportion of clinical medicine majored students who would choose psychiatry as a career (0.69%). Personal interest (59.61%), suggestions from family members (27.96%), and experiencing mental problems (23.19%) were main reasons for choosing psychiatry major at matriculation. Personal interest (odds ratio [OR] = 2.12, 95% confidence interval [CI] = 1.87–2.40), experiencing a psychiatry clerkship (OR = 1.99, 95% CI = 1.28–3.08), being female (OR = 1.50, 95% CI = 1.30–1.68), experiencing mental problems (OR = 1.33, 95% CI = 1.28–1.56), and suggestions from family members (OR = 1.25, 95% CI = 1.08–1.46) correlated positively with students’ choice of psychiatry as career. Students who lacked psychiatry knowledge (OR = 0.49, 95% CI = 0.29–0.85) or chose psychiatry because of lower admission scores (OR = 0.80, 95% CI = 0.63–0.97) were less likely to choose psychiatry as a career.

**Conclusion:**

More than half of psychiatry majored medical school students planned to choose psychiatry as their career, whereas very few students in the clinic medicine major would make this choice. Increasing students’ interest in psychiatry, strengthening psychiatry clerkships, and popularizing psychiatric knowledge are modifiable factors to increase the psychiatry career intention. The extent to which medical students’ attitudes toward psychiatry can be changed through medical school education and greater exposure to psychiatry will need further investigation.

**Supplementary Information:**

The online version contains supplementary material available at 10.1186/s12909-021-02622-x.

## Background

In China, mental health problems are prominent. The disease burden of mental disorders in China accounts for 13% of all non-communicable disease burden domestically and 17% of the global mental disorder burden [1]. In 2017, for every 100,000 people in the population, there were only 2.19 psychiatrists (including assistant practitioners) in China compared with 13.06 psychiatrists in high-income countries [2, 3]. Among registered psychiatrists, 40% had only a technical school degree or even no academic degree [4]. Historically, medical education in psychiatry has been a challenge in China. In the 1960s, there were only three medical schools in China that recruited undergraduates to the psychiatry specialty. Based on statistics from the Psychiatry Teaching Steering Committee, the rate of a psychiatry career choice was around 30%. A few studies have evaluated attitudes toward psychiatry and mental illness among Chinese medical students [5–10], but studies on psychiatry as a career choice have been scarce. One study showed that approximately 1.6% of fourth-year medical students in Xiangya Medical School chose psychiatry as a career [9].

In recent years, the Chinese government has implemented a series of initiatives to improve mental health services [11]. As a part of the Healthy China 2030 Plan, countermeasures to increase the recruitment of psychiatrists were implemented by the Chinese Ministry of Education, including the further establishment of psychiatry undergraduate education after 2014 and the Psychiatry Teaching Steering Committee to guide psychiatry education. In many medical colleges, psychiatry has been established as a subspecialty in clinical medicine to promote the recruitment of psychiatrists. Students could choose the psychiatry specialty at the beginning of medical school and graduate with a bachelor’s degree in psychiatry. Students in the psychiatry specialty take the same courses as students in the clinical medicine specialty from the first year to the third year. In addition, they are required to take courses of psychiatry and psychology in a systematic way in the third to fifth years and receive both clinical training and psychiatry clerkship in the fourth and fifth years. However, students of clinical medicine degree take psychiatry as an elective course with psychiatry externship but without psychiatry clerkship. As of 2019, the psychiatry specialty was established in 31 medical schools [2], of which 30 had recruited 6271 students in the psychiatry specialty.

Still unknown, however, is the percentage of students in the psychiatry specialty who are expected to become psychiatrists after graduation. Furthermore, to maintain a sustainable high-quality source of psychiatrists, it is important to learn why students choose the psychiatry specialty at the beginning of their studies in medical school and the factors that influence their choice to become a psychiatrist after graduation. Few studies have explored the career choice of students in the psychiatry specialty and its influential factors.

The present study (1) explored the characteristics of the career choice of undergraduate students in the psychiatry specialty and (2) identified factors that influence their career choice. We sought to generate data that would support psychiatry medical education reform and recruitment policies for psychiatrists in China.

## Methods

### Participants and procedure

Medical students who majored in psychiatry and clinical medicine from all 31 medical universities with a psychiatry specialty in China were invited to participate in this study from May to October of 2019. Students in six universities were not included in the study: three universities refused to participate, two did not receive ethical approval, and one did not start to enroll students at the appointed investigation period. In order to enlarge the sample size of students majoring in clinical medicine, one more university (Guizhou Medical university) with clinical medicine specialty were recruited. Finally, students majoring in psychiatry and clinical medicine from 26 medical universities take part in the investigation. All first to fifth year students majoring in psychiatry (students of year 2014 to year 2018) participated in this survey during May 15 and August 30, 2019. The year 2019 freshmen, who were newly enrolled in medical universities in year 2019 and did not receive any medical education when this survey took place were investigated from September 1 to October 24, 2019. Students in clinical medicine were included as a comparison group, who were cluster-sampled randomly by classes from the same school and grade as students in the psychiatry major. A total of 12,696 questionnaires were sent to students, and 9054 students completed the questionnaire (71.31% response rate). Of these 9054 students, 8467 (93.52%) were validated respondents (4610 psychiatry students and 3857 clinical medicine students). The number and proportion of respondents from each school are shown in Online Resource 1.

The participants were well informed of this survey by the investigators of each subcenter and signed written informed consent forms before they had access to the electronic questionnaire. The students could decide to complete the questionnaire either on their mobile phone (by scanning a QR code) or on a computer (by logging in to a website). The questionnaire took approximately 15 min to complete. The data were collected anonymously. Each student was asked to answer the questions on the day they received the survey link. We counted the progress of survey completion every day and gave feedback to investigators in the subcenters. The data were collected and maintained by the H6WORLD Clinical Research Data Platform (https://h6world.cn), which is supported by the Peking University National Engineering Research Center. The present study was approved by the ethics committee at Peking University Sixth Hospital.

### Measurements

#### Sociodemographics

The sociodemographic information included age, gender, grade (classified into three levels: freshman, first year to third year as low grade, fourth and fifth year as high grade), learning stage (basic knowledge learning, externship [shadowing the attendings in the hospital] and clerkship), family history of mental disorders, history of using psychiatry services, family economic status, and living area.

#### Specialty choice and influential factors

The specialty choice for psychiatry and related factors were evaluated in two stages. The first stage was the choice for the psychiatry specialty at matriculation. The second stage was the choice of psychiatry as a career. Students in the psychiatry major were asked how they would rank the choice of psychiatry when they completed specialty applications at matriculation. For career choice, the respondents could choose a favorite specialty among 26 specialties in the hospital or “Undecided” or “I don’t plan to practice medicine.”

To reveal the reasons for choosing and rejecting the psychiatry specialty at matriculation, students in the psychiatry major were asked the question, “Why did you choose psychiatry at matriculation?” Students in clinical medicine were asked, “Why didn’t you choose psychiatry at matriculation?” Answers to these questions were sought as a free-text response. Some respondents gave a single reason, but most of them gave several reasons. Based on the responses, a coding scheme was developed to reflect the main themes that were raised by the respondents (for example statements, see Online Resource 2). Two authors independently coded all of the reasons given, by theme, and compared each other’s coding. These reasons of choosing psychiatry were used as independent variables in the career choice analysis. Moreover, the influence of demographic characteristics on the psychiatry specialty choice at matriculation was also assessed.

The potential influential factors for the psychiatry career choice were assessed among medical students who chose the psychiatry major at matriculation with a series of questions. First, the participants were asked about their general considerations for a career path (e.g., potential income, work-life balance, content of the specialty, etc.). Detailed questions are shown in Online Resource 3. Second, participants’ attitudes toward the psychiatry specialty were evaluated using the 22-item Attitudes Toward Psychiatry (ATP-22) scale, which has been validated and used in Chinese medical students [5, 6]. Third, participants’ self-stigma about mental illness was rated using the 15-item Stigma Scale, which was adapted from the study by Schwenk [12], translated into Chinese and tested for internal consistency and reliability (Cronbach’s α coefficient = 0.74) in medical students [13]. All of the items were added to form a total score (ranging from 0 to 15). Higher scores indicated a higher level of stigma. Fourth, macroscopic factors, such as regional economic level, the capacity of mental health services, and other relevant information from China Health Statistics Yearbook 2018 [14], were also collected.

Survey items (Online Resource 3) that were coded using a Likert scale were recoded as dichotomous variables, and the answers were coded into important and other. For example, for the question, “When thinking about your career specialty after medical school, how important are the following considerations?” answers of very important and partially important were coded as important. Other answers were code as other.

### Data analysis

Frequencies and means with standard deviations were calculated for descriptive data, and *t*-tests were used to compare mean values for continuous variables. The *χ*^*2*^ test was used for categorical variables. Two authors reviewed the survey items. Based on a literature review [7, 9, 13, 15–37], they identified 36 student-level factors, including demographic factors, matriculation reasons, and experiential items, that were hypothesized to be associated with the psychiatry career choice (Online Resource 4). Logistic regression analysis was used as the original analysis for variable screening, with the selection of either psychiatry or any other specialty as a career choice as the binary dependent variable. Variables with *p* <  0.05 in univariable logistic regression model were selected for the multivariable logistic regression model. A total of 21 significant variables that were identified in the bivariate analysis (Online Resource 4) were then entered into a multivariable model using a backward stepwise procedure, and finally 15 variables remained. All of the analyses were performed using SPSS 26.0 software (SPSS, Chicago, IL, USA).

## Results

### Demographic characteristics

The mean age of the respondents in psychiatry and clinical medicine was 20.36 ± 1.41 and 19.90 ± 1.39 years old, respectively. Females accounted for 62.22% of the psychiatry major respondents and 53.34% of the clinical medicine respondents. Half of the respondents lived in the city and had a family income of 3000–10,000 RMB per month. Most of the students were in the basic knowledge learning stage. Detailed demographic information is listed in Table 1.

**Table 1:** Demographic characteristics of 8467 respondents from 26 medical schools in China, 2019

	Psychiatry ***n*** = 4610	Clinical medicine ***n*** = 3857
**Age (years) (mean [SD])**	20.36 (1.41)	19.90 (1.39)
**Female (no. [%])**	2868 (62.22)	2061 (53.34)
**Family monthly income (no. [%])**		
< 1000 RMB	194 (4.21)	227 (5.89)
1000–2999 RMB	843 (18.29)	790 (20.48)
3000–4999 RMB	1180 (25.60)	1027 (26.63)
5000–9999 RMB	1384 (30.02)	1077 (27.92)
10,000–14,999 RMB	564 (12.23)	406 (10.53)
15,000–20,000 RMB	203 (4.40)	153 (3.97)
> 20,000 RMB	226 (4.90)	126 (3.27)
**Family living area (no. [%])**		
Village	1349 (29.26)	1290 (33.45)
Town	955 (20.72)	890 (23.07)
City	2290 (49.67)	1626 (42.16)
**Undergraduate year (no. [%])**		
Freshman	946 (20.52)	1721 (44.62)
Low grade	3148 (68.27)	1979 (51.31)
High grade	517 (11.21)	157 (4.07)
**Learning stage (no. [%])**		
Basic knowledge learning	3949 (85.66)	3613 (93.67)
Externship	436 (9.50)	170 (4.41)
Clerkship	225 (4.88)	74 (1.92)
**Learning hours per day (mean [SD])**	6.18 (5.78)	6.67 (3.87)
**History of using psychiatry service (no. [%])**	114 (2.47)	49 (1.27)
**Family history of mental disorders (no. [%])**	443 (10.63)	231 (6.37)
**ATP-22 score (mean [SD])**	55.80 (8.79)	52.12 (11.84)
**Stigma score (mean [SD])**	8.26 (3.29)	7.94 (3.38)

*SD* standard deviation, *ATP* Attitudes Toward Psychiatry

### Reasons for choosing and not choosing the psychiatry specialty at matriculation

The results indicated that 59.61% of the students who chose psychiatry and 58.93% of the students who rejected the psychiatry specialty at matriculation made their choice based on “personal interest” (Table 2). A total of 27.96% of the students chose and 7.81% of the students did not choose the psychiatry specialty because of the suggestions or influence of their parents or other family members. A total of 23.19% of the students chose the psychiatry specialty because they hoped to help themselves or other people in need because of their experience with negative mental health status or being a witness to people with mental health problems. Moreover, 11.28% of the students chose the psychiatry specialty because of their lower scores at matriculation, which only met the requirement for the psychiatry specialty.

**Table 2:** Reasons for choosing and rejecting the psychiatry specialty at matriculation

Reason	Choosing psychiatry (Psychiatry major) ***n*** = 4610 (no. [%])	Objecting psychiatry (Clinical medicine major) ***n*** = 3857 (no. [%])
**Personal interest**	2748 (59.61)	2273 (58.93)
**Family’s suggestion or influence**	1289 (27.96)	301 (7.81)
**Experience of mental problems of oneself or others**	1069 (23.19)	–
**Low admission score of psychiatry**	520 (11.28)	17 (0.99)
**Lack knowledge of psychiatry**	72 (1.56)	83 (2.15)
**Positive/negative attitudes toward psychiatry**	30 (0.65)	163 (4.23)
**School**	11 (0.24)	117 (3.06)
**Other reasons**	705 (15.29)	443 (11.67)

The influence of demographic characteristics on the psychiatry specialty choice at matriculation is shown in Table 3. Higher family income was the most positively correlated factor of choosing psychiatry at matriculation (odds ratio [OR] = 2.08, 95% confidence interval [CI] = 1.52–2.84, comparing > 20,000 RMB/month and < 1000 RMB/month). The female gender (OR = 1.47, 95% CI = 1.34–1.61), living in a city (OR = 1.21, 95% CI = 1.08–1.35), a history of utilizing mental health services (OR = 1.55, 95% CI = 1.09–2.20), and a family history of mental disorders (OR = 1.54, 95% CI = 1.30–1.83) were positively associated with choosing the psychiatry specialty at matriculation.

**Table 3:** Influence of demographic characteristics on psychiatry specialty choice at matriculation

Demographic characteristic	***n***	Choice (no. [%])	OR (95% CI)	***p***
**Gender**				
Male	3538	848 (24.00)	reference	
Female	4929	1733 (35.20)	1.47 (1.34–1.61)	< 0.001
**Family living area**				
Village	2638	741 (28.10)	reference	
Town	1845	544 (29.50)	0.99 (0.87–1.12)	0.879
City	3916	1296 (33.10)	1.21 (1.08–1.35)	0.001
**Family monthly income**				
< 1000 RMB	421	101 (24.00)	reference	
1000–2999 RMB	1633	447 (27.40)	1.32 (1.05–1.65)	0.017
3000–4999 RMB	2206	695 (31.40)	1.41 (1.13–1.76)	0.002
5000–9999 RMB	2461	782 (31.80)	1.54 (1.24–1.93)	< 0.001
10,000–14,999 RMB	970	320 (33.00)	1.60 (1.24–2.04)	< 0.001
15,000–20,000 RMB	356	116 (32.60)	1.49 (1.10–2.02)	0.010
> 20,000 RMB	352	120 (34.10)	2.08 (1.52–2.84)	< 0.001
**History of using psychiatry services**				
No	8235	2508 (30.50)	reference	
Yes	163	73 (44.80)	1.55 (1.09–2.20)	0.013
**Family history of mental disorders**				
No	7792	2321 (29.80)	reference	
Yes	674	260 (38.60)	1.54 (1.30–1.83)	< 0.001

### Career choice for the psychiatry specialty during medical education

The results showed that 56.67% (2556/4510) of the students in the psychiatry specialty chose psychiatry as their career (Table 4). The rates of choice among males and females were 48.16% (839/1742) and 59.87% (1717/2868), respectively. Compared with freshmen (471/946, 49.74%), students in high grades (337/517, 65.18%) and students in low grades (1749/3146, 55.58%) had a higher preference for choosing psychiatry as a career (*χ*^*2*^ = 32.58, *p* <  0.001, significant difference between groups after Bonferroni correction). Only 0.69% (26/3792) of the students in clinical medicine considered psychiatry as a career. Psychiatry-related subjects, such as neurology (11.91% vs. 0.84%, *χ*^*2*^ = 394.92, *p* <  0.001) and neurosurgery (5.17% vs. 2.48%, *χ*^*2*^ = 48.53, *p* <  0.001), were also more popular among psychiatry major students compared with students in clinical medicine.

**Table 4:** Top 10 career choice among students in psychiatry major and clinical medicine major (listed as descending selection rate in each group)

Specialty	Psychiatry 4510 (no. [%])	Specialty	Clinical medicine 3792 (no. [%])
Psychiatry	2556 (56.67)	Undecided	989 (26.08)
Undecided	555 (12.31)	Internal Medicine	589 (15.53)
Neurology	537 (11.91)	Surgery	507 (13.37)
Neurosurgery	233 (5.17)	Obstetrics and Gynecology	247 (6.51)
Internal Medicine	150 (3.33)	Orthopedic Surgery	241 (6.36)
Surgery	93 (2.06)	Emergency Medicine	204 (5.38)
Anesthesiology	54 (1.20)	Thoracic Surgery	119 (3.14)
Obstetrics and Gynecology	49 (1.09)	Ophthalmology	110 (2.90)
Emergency Medicine	45 (1.00)	Pediatrics	97 (2.56)
Don’t plan to practice medicine	39 (0.86)	Neurosurgery	94 (2.48)

Except for undecided students (*n* = 989, 26.08%), the most preferred specialty choice among clinical medicine students was internal medicine (*n* = 589, 15.53%), followed by surgery (*n* = 507, 13.37%), obstetrics and gynecology (*n* = 247, 6.51%), and orthopedic surgery (*n* = 241, 6.36%).

### Factors influencing psychiatry career choice

Among students who chose the psychiatry specialty at matriculation, several factors were positively associated with choosing psychiatry as a career (Table 5), including students’ personal interest in psychiatry (OR = 2.12, 95% CI = 1.87–2.40), experiencing a psychiatry clerkship (OR = 1.99, 95% CI = 1.28–3.08), experiencing mental problems oneself or with others (OR = 1.33, 95% CI = 1.28–1.56), influence of family members (OR = 1.25, 95% CI = 1.08–1.46), female (OR = 1.50, 95% CI = 1.30–1.68), high grade (OR = 1.13, 95% CI = 1.07–1.21), reporting work-life balance (OR = 1.15, 95% CI = 1.04–1.26) and content of specialty (OR = 1.10, 95% CI = 1.00–1.19) as important consideration for career path, favorable attitude toward psychiatry (OR = 1.03, 95% CI = 1.02–1.04), and self-stigma of mental disorders (OR = 1.05, 95% CI = 1.03–1.07). Entering the psychiatry major without knowing about psychiatry (OR = 0.49, 95% CI = 0.29–0.85) or because of a lower admission score (OR = 0.80, 95% CI = 0.63–0.97) was negatively associated with choosing psychiatry as a career. Other negative factors included level of educational debt (OR = 0.90, 95% CI = 0.84–0.98) and leadership potential (OR = 0.87, 95% CI = 0.80–0.94).

**Table 5:** Multivariable regression analysis of influential factors for psychiatry career choice among psychiatry major students

	***n***	Choice (no. [%])	OR (95% CI)	***p***
**Demographic characteristic**				
**Gender**				
Male	1742	839 (48.20)	reference	
Female	2868	1716 (59.90)	1.50 (1.30–1.68)	< 0.001
**Grade**				
Freshman	946	470 (49.70)	reference	
Low grade	3146	1748 (55.60)	1.11 (0.78–1.57)	0.507
High grade	517	337 (65.20)	1.13 (1.07–1.21)	< 0.001
**Learning stage**				
Basic knowledge learning	3949	2106 (53.30)	reference	
Externship	436	296 (67.90)	1.74 (1.32–2.31)	< 0.001
Clerkship	225	153 (68.00)	1.99 (1.28–3.08)	0.002
**Reasons for choosing psychiatry major at matriculation**				
**Personal interest**				
No	1862	828 (44.50)	reference	
Yes	2748	1775 (64.60)	2.12 (1.87–2.40)	< 0.001
**Experience of mental problems of oneself or others**				
No	3541	1846 (53.50)	reference	
Yes	1069	677 (63.30)	1.33 (1.28–1.56)	0.001
**Family’s suggestion or influence**				
No	3321	1934 (55.70)	reference	
Yes	1289	691 (61.37)	1.25 (1.08–1.46)	0.004
**Lack knowledge of psychiatry**				
No	4538	2534 (55.80)	reference	
Yes	72	21 (29.60)	0.49 (0.29–0.85)	0.010
**Low admission score of psychiatry**				
No	4090	2323 (56.80)	reference	
Yes	520	220 (42.30)	0.80 (0.63–0.97)	0.045
**General considerations when thinking about career specialty**				
**Work-life balance**				
Not important	876	486 (55.50)	reference	
Important	3623	2060 (56.90)	1.15 (1.04–1.26)	0.006
**Content of specialty**				
Not important	922	483 (52.40)	reference	
Important	3577	2063 (57.70)	1.10 (1.00–1.19)	0.041
**Level of educational debt**				
Not important	2270	1360 (59.90)	reference	
Important	2229	1186 (53.20)	0.90 (0.84–0.98)	0.006
**Length of residency training**				
Not important	1685	1000 (59.30)	reference	
Important	2813	1546 (54.90)	0.93 (0.85–1.01)	0.226
**Leadership potential**				
Not important	2261	1333 (59.00)	reference	
Important	2244	1219 (54.30)	0.87 (0.80–0.94)	0.001
**Stigma scale (continuous variable)**			1.05 (1.03–1.07)	< 0.001
**ATP-22 (continuous variable)**			1.03 (1.02–1.04)	< 0.001

*ATP* Attitudes Toward Psychiatry

Regional gross domestic product per capita positively correlates with the number of health workers and psychiatrists. However, neither the economic factor nor the mental health service capacity factor was correlated with choosing psychiatry as a career (Online Resource 5).

For clinical medicine, logistic regression was not performed because of the low statistical power of the limited positive samples. A stable and secure future (23, 88.46%), job availability (23, 88.46%), and work-life balance (21, 80.77%) were the most stated reasons among the 26 students who would choose psychiatry as a career.

## Discussion

The present study explored the characteristics and related influential factors of career choice among students in psychiatry and clinical medicine specialties in China. The motivation for majoring psychiatry at the point of matriculation, including personal interest, influence of family members, and experiences of mental problems, were pre-medical school factors that had a strong influence on choosing psychiatry as a career. During medical school, medical education, especially psychiatry clerkships and the lifestyle that is associated with a psychiatry career, were attractive to students and influenced their decision to pursue psychiatry as a career. Increasing mental health knowledge and interest among high school students, strengthening psychiatry clerkships, and reducing the stigma of mental illness in the community are modifiable factors that should be considered in education reform. The present study provided information to improve the psychiatry education system and maintaining a stable pool of psychiatry professionals.

After years of cultivating psychiatry as a specialty, the number of registered psychiatrists in China increased from 1.54 per 100,000 in 2010 to 2.19 per 100,000 in 2017. The present results showed that the psychiatry career choice among students who majored in psychiatry increased to 56%, which was only 30% in the 1960s. However, nearly half of the students did not plan to choose psychiatry as a career. Less than 0.7% of the students with a clinical medicine major would choose to work as a psychiatrist. Targeted efforts both before and during medical school should be made to increase the intention of choosing the psychiatry specialty.

### Personal factors

Previous studies suggested that one of the most often stated reasons by the students for considering psychiatry at matriculation and as a career was their sincere interest in this field [18, 38]. Hence, building up students’ interest in psychiatry and giving more chance to students with real inherent interest in psychiatry for admission would be essential strategies for increasing the number of students who ultimately pursue a career in psychiatry [39].

In the present study, work-life balance was positively correlated with the psychiatry career choice among students who majored in psychiatry and was also highly valued among students who majored in clinical medicine but considered psychiatry as a career. Balancing work and family has become increasingly important for medical students when considering about a profession [28]. Psychiatry is a specialty with controllable lifestyle factors, such as control over work hours, the perceived number of nights on call, and adequate time to pursue personal activities [40, 41]. The students’ value of work-life balance positively correlated with the psychiatry career choice [22], and controllable lifestyle could explain 55% of the variability in specialty preference among medical students [28]. Additionally, work-family conflicts (i.e., inter-role conflicts) impacted mental and somatic health [42]. With regard to gender, females were more likely to choose psychiatry [38, 39]. Females were more likely to seek a balance between their professional and private lives [43] and were less likely to expect to earn a high income [44]. Therefore, the advantageous characteristics of the psychiatry profession should be promoted so that more students may be attracted to psychiatry.

### Mental illness experience and self-stigma

Mental health knowledge is helpful, especially among those with mental health problems and those who hope to help others [45, 46]. The psychiatry curriculum naturally involves the enrichment of mental health knowledge. The present study found that students who suffered from mental health problems or had family members with mental disorders were more likely to choose the psychiatry specialty, which was consistent with previous research [39]. Moreover, such experience could be a motivational factor to become a psychiatrist to provide support for both oneself and others in need. Meanwhile, the self-stigma of mental illness was positively correlated with the psychiatry career choice. The source of self-stigma of mental illness among medical students is mainly based on personal experience, social stereotypes [33], and school environment [12]. Fear of professional sanctions may negatively impact self-confidence, making them feel less competitive [35] and more reluctant to disclose their mental status on licensure and medical staff applications [47]. It may also lead to inappropriate and possibly dangerous approaches of seeking help. Current medical education should focus on student psychiatric knowledge and specific measures to reduce students’ self-stigma.

### Family and school influence

Family comments and advice, mainly from parents, played an important role in students’ choice of specialty. Approximately 8% of clinical medicine students who were interested in psychiatry failed to choose psychiatry as a major at matriculation because of their parents’ objections. The values and career views of older generations are significantly different from those of the 21st century [48]. While in the psychiatry major, families’ suggestions were a supportive factor for choosing psychiatry at matriculation and as a career. Students with this characteristic mainly reported that they had family members who had favorable experiences in psychiatry, such as achieving better mental health literacy and having controllable lifestyles. Popularizing psychiatry and encouraging family members to participate in recruitment programs may promote the choice of psychiatry as a specialty.

Previous studies reported a positive effect of favorable attitudes toward psychiatry on the career choice of psychiatry [17, 18, 20, 21]. The present results among medical students in China are consistent with previous studies. The experience of clerkships and other opportunities to interact with patients [19] increases the understanding of psychiatry and fosters the ability to communicate with patients with mental illness [48], improves attitudes about psychiatry and mental illness [49], and consequently encourages more students to choose the psychiatry specialty as a career. Meanwhile, psychiatrists who worked with students play an important role in developing creative and imaginative psychiatry programs [19]. In the present study, students who had contact with patients with mental illness and finished clinical rotations (e.g., clerkships) in psychiatry were more likely to choose psychiatry as a career, which is consistent with many previous studies [10, 20–22, 39]. Medical schools should provide psychiatry clerkships to promote students’ interest in psychiatry. Further research is also needed to explore the relationship between psychiatry clerkship characteristics (e.g., preceptor and mode of teaching) and students’ career choice.

Lower admission scores were also associated with choosing the psychiatry specialty at matriculation, but it might not be associated with choosing psychiatry after graduation. In the present study, among students with lower scores upon entrance to medical school, only about a quarter of them would like to pursue psychiatry as a career, whereas half of them changed to other specialties, such as neurology, neurosurgery, internal medicine, and surgery. Thus, lower college admission requirements (e.g., lower admission scores) is not a useful measure for improving the recruitment of students to the psychiatry specialty.

### Socioeconomic status and medical health service factors

Family income status was a predictive factor for medical students’ career choice [50]. According to the data of National Bureau of Statistics of China [51], the per capita disposable income of Chinese residents was about 30,000 RMB per year, 2500 RMB per month in 2019. The average income for a family of three was about 7500 RMB per month. In the present study, students with a high family income over 20,000 RMB per month were twice as likely to choose psychiatry at the beginning of medical school compared with those with a low family income of less than 1000 RMB per month. Socioeconomic factors may have a profound impact on medical students’ career choice. A long-term observational study of a cohort of medical students reported a change in career choice for psychiatry as socioeconomic status changed over time [22–24, 52]. However, in the present study, none of the socioeconomic factors or mental health service capacity factors significantly correlated with the career choice of psychiatry (Online Resource 5). The possible reason for this finding could be that some relatively underdeveloped areas may have special support programs and employment promotion policies for students’ recruitment to the psychiatry specialty, which makes the job possibility much greater than in developed provinces. However, psychiatry students’ employment choice is consistent nationwide in China. The economic level of the province where the school is located does not necessarily represent the target location of the vocation. Future studies should explore the relationship between more specific socioeconomic factors and the career choice of psychiatry, including average income of the local psychiatry department and investment in psychiatry.

To meet the public demand for mental health services, teams of mental health professionals, including psychiatrists, should be strengthened [3, 53]. It is challenging to establish a strategy to recruit more students, especially outstanding students in psychiatry and clinical medicine specialties, to study and work in the mental health field. The following strategies should be considered. First, the national psychiatry education and training system should be strengthened to include enrichment activities in the undergraduate psychiatry curriculum and clerkships. Second, mental health services should be emphasized in the medical health services system so that more students in psychiatry and clinical medicine specialties might be encouraged to work in the mental health field. Third, given the lack of mental health knowledge among the general population, education on mental health and psychological problems should be improved in high schools.

### Limitations

The present study has limitations. First, this was a cross-sectional survey, and the reasons for the specialty choice at matriculation may have been influenced by recall bias. The extent to which views about psychiatry among psychiatry students can be modified by the medical school education and greater exposure to psychiatry needs further investigation. Longitudinal follow-up studies should be conducted to explore students’ career choice after they graduate from medical school. Second, the samples were designed to be equally distributed in the two majors. However, because of different response rates, the samples included fewer freshmen and more first-year to fifth-year students in the psychiatry major compared with clinical medicine. The hierarchical analysis was conducted by grade (Supplemental Digital Content 6). The results of freshmen and low-grade students was consistent with the total population. However, because of the relatively small number of respondents, only one variable (family living area) was significant in high-grade students. Third, the choice of psychiatry as a career was influenced by personal factors, such as sex, interest in the specialty, lifestyle, family influences, medical education, specialty content, and stigma of mental illness. We collected these data and explored the impact on students’ choices. However, the underlying relationships and mechanisms of these factors are still unknown. Further studies are needed to address these limitations.

## Conclusions

More than half of the students in the psychiatry specialty planned to choose psychiatry as their long-term career in medical school, whereas students in the clinical medicine specialty seldom did so. Increasing mental health knowledge and interest among high school students, strengthening psychiatry clerkships, removing barriers from families and schools, and reducing the stigma of mental illness are crucial for the development of mental health professionals.

## Supplementary Information

**Additional file 1.**

## Data Availability

Our data and materials will not be available at present.

## Abbreviations

OR: Odds ratio
CI: Confidence interval
ATP-22: 22-item Attitudes Toward Psychiatry scale
SD: Standard deviation

## References

[1] Charlson FJ, Baxter AJ, Cheng HG, Shidhaye R, Whiteford HA (2016). The burden of mental, neurological, and substance use disorders in China and India: a systematic analysis of community representative epidemiological studies. Lancet..

[2] Que J, She L, Liu J, Sun Y, Bao Y, Lu L (2019). The development of mental hospitals in China from 2002 to 2016. Chin J Psychiatry.

[3] Que J, Lu L, Shi L (2019). Development and challenges of mental health in China. Gen Psychiatr.

[4] Liu C, Chen L, Xie B, Yan J, Jin T, Wu Z (2013). Number and characteristics of medical professionals working in Chinese mental health facilities. Shanghai Arch Psychiatry.

[5] Liu T, Cao Y, Zhang Y, Xue Z (2004). Pilot study of attitudes toward psychiatry among medical students. Nerv Dis Mental Hyg.

[6] Wang X, Xiang X, Hao W, T. L (2011). A attitude toward psychiatry among medical students. J Cent South Univ (Med Sci).

[7] Zhong J, Zheng L, Chen X, Gao Q, Zhang B, Wang W (2016). Influencing factors on choosing psychiatry as a career: an exploration in Chinese University students. Psychiatr Q.

[8] Zhu Y, Zhang H, Yang G, Hu X, Liu Z, Guo N, He H, Sun B, Rosenheck R (2018). Attitudes towards mental illness among medical students in China: impact of medical education on stigma. Asia Pac Psychiatry.

[9] Wang X, Xiang X, Hao W, Liu T (2013). Attitudes toward psychiatry as a prospective career among medical students in their pre-clinical year in China- a pilot study. PLoS One.

[10] Shen Y, Dong H, Fan X, Zhang Z, Li L, Lv H, Xue Z, Guo X (2014). What can the medical education do for eliminating stigma and discrimination associated with mental illness among future doctors? Effect of clerkship training on chinese students' attitudes. Int J Psychiatry Med.

[11] Liu J, Ma H, He YL (2011). Mental health system in China: history, recent service reform and future challenges. World Psychiatry.

[12] Schwenk TL, Davis L, Wimsatt LA (2010). Depression, stigma, and suicidal ideation in medical students. JAMA..

[13] Huang W, Que J, Liu Z, et al. The anticipated stigma to mental problem among medical students. Chin J Drug Dependece. 2020; Accepted.

[14] National Health Commission (2018). China Health Statistics Yearbook.

[15] Are C, Stoddard HA, O'Holleran B, Thompson JS (2012). A multinational perspective on "lifestyle" and other perceptions of contemporary medical students about general surgery. Ann Surg.

[16] Pailhez G, Bulbena A, Coll J, Ros S, Balon R (2005). Attitudes and views on psychiatry: a comparison between Spanish and U.S. medical students. Acad Psychiatry.

[17] Feifel D, Moutier CY, Swerdlow NR (1999). Attitudes toward psychiatry as a prospective career among students entering medical school. Am J Psychiatry.

[18] Farooq K, Lydall GJ, Malik A, Ndetei DM, Bhugra D (2014). Why medical students choose psychiatry - a 20 country cross-sectional survey. BMC Med Educ.

[19] Ramos MA, Rotenstein LS, Mata DA (2016). Student-run free clinics: the USA's psychiatry recruitment solution?. Lancet Psychiatry.

[20] McParland M, Noble LM, Livingston G, McManus C (2003). The effect of a psychiatric attachment on students' attitudes to and intention to pursue psychiatry as a career. Med Educ.

[21] Economou M, Kontoangelos K, Peppou LE, Arvaniti A, Samakouri M, Douzenis A, Papadimitriou GN (2017). Medical students' attitudes to mental illnesses and to psychiatry before and after the psychiatric clerkship: training in a specialty and a general hospital. Psychiatry Res.

[22] Goldenberg MN, Williams DK, Spollen JJ (2017). Stability of and factors related to medical student specialty choice of psychiatry. Am J Psychiatry.

[23] Goldacre MJ, Fazel S, Smith F, Lambert T (2013). Choice and rejection of psychiatry as a career: surveys of UK medical graduates from 1974 to 2009. Br J Psychiatry.

[24] Newton DA, Grayson MS (2003). Trends in career choice by US medical school graduates. JAMA..

[25] Funston G, Young A (2013). UK medical students, debt, and career choices. Lancet..

[26] McLean AL, Piper R, Carmichael J, Qureshi Z, Ma A, Russell CD (2013). UK medical students, academia, and the financial crisis. Lancet..

[27] Lee CW (2013). Gender difference and specialty preference in medical career choice. Korean J Med Educ.

[28] Dorsey ER, Jarjoura D, Rutecki GW (2003). Influence of controllable lifestyle on recent trends in specialty choice by US medical students. JAMA..

[29] Newton DA, Grayson MS, Thompson LF (2005). The variable influence of lifestyle and income on medical students' career specialty choices: data from two U.S. medical schools, 1998-2004. Acad Med.

[30] Levine RB, Mechaber HF, Reddy ST, Cayea D, Harrison RA (2013). "a good career choice for women": female medical students' mentoring experiences: a multi-institutional qualitative study. Acad Med.

[31] Lambert EM, Holmboe ES (2005). The relationship between specialty choice and gender of U.S. medical students, 1990-2003. Acad Med.

[32] Clinite KL, DeZee KJ, Durning SJ (2014). Lifestyle factors and primary care specialty selection: comparing 2012-2013 graduating and matriculating medical students' thoughts on specialty lifestyle. Acad Med.

[33] Morris E, Hippman C, Murray G, Michalak EE, Boyd JE, Livingston J, Inglis A, Carrion P, Austin J (2018). Self-stigma in relatives of people with mental illness scale: development and validation. Br J Psychiatry.

[34] Petkari E, Masedo Gutierrez AI, Xavier M, Moreno KB (2018). The influence of clerkship on students' stigma towards mental illness: a meta-analysis. Med Educ.

[35] Chew-Graham CA, Rogers A, Yassin N (2003). 'I wouldn't want it on my CV or their records': medical students' experiences of help-seeking for mental health problems. Med Educ.

[36] Cleland JA, Johnston P, Watson V, Krucien N, Skåtun D (2017). What do UK medical students value most in their careers? A discrete choice experiment. Med Educ.

[37] Gutmann L, Cahill C, Jordan JT, Gamaldo CE, Santini V, Ali I, Soni M, Wilson RB, Said R, Czeisler BM, Smith AG (2019). Characteristics of graduating US allopathic medical students pursuing a career in neurology. Neurology..

[38] Warnke I, Gamma A, Buadze M, Schleifer R, Canela C, Strebel B, Tényi T, Rössler W, Rüsch N, Liebrenz M (2018). Predicting medical Students' current attitudes toward psychiatry, interest in psychiatry, and estimated likelihood of working in psychiatry: a cross-sectional study in four European countries. Front Psychiatry.

[39] Shields G, Ng R, Ventriglio A, Castaldelli-Maia J, Torales J, Bhugra D (2017). WPA position statement on recruitment in psychiatry. World Psychiatry.

[40] Schwartz RW, Haley JV, Williams C, Jarecky RK, Strodel WE, Young B, Griffen WO (1990). The controllable lifestyle factor and students' attitudes about specialty selection. Acad Med.

[41] Schwartz RW, Jarecky RK, Strodel WE, Haley JV, Young B, Griffen WO (1989). Controllable lifestyle: a new factor in career choice by medical students. Acad Med.

[42] Jerg-Bretzke L, Limbrecht-Ecklundt K, Walter S, Spohrs J, Beschoner P (2020). Correlations of the "work-family conflict" with occupational stress-a cross-sectional study Among University employees. Front Psychiatry.

[43] Yamazaki Y, Uka T, Marui E (2017). Professional fulfillment and parenting work-life balance in female physicians in basic sciences and medical research: a nationwide cross-sectional survey of all 80 medical schools in Japan. Hum Resour Health.

[44] Bergquist SR, Duchac BW, Schalin VA, Zastrow JF, Barr VL, Borowiecki T (1985). Perceptions of freshman medical students of gender differences in medical specialty choice. J Med Educ.

[45] Waqas A, Naveed S, Makhmoor A, Malik A, Hassan H, Aedma KK (2020). Empathy, experience and cultural beliefs determine the attitudes towards depression among Pakistani medical students. Community Ment Health J.

[46] Ibrahim N, Amit N, Shahar S, Wee LH, Ismail R, Khairuddin R, Siau CS, Safien AM (2019). Do depression literacy, mental illness beliefs and stigma influence mental health help-seeking attitude? A cross-sectional study of secondary school and university students from B40 households in Malaysia. BMC Public Health.

[47] Center C, Davis M, Detre T, Ford DE, Hansbrough W, Hendin H, Laszlo J, Litts DA, Mann J, Mansky PA, Michels R, Miles SH, Proujansky R, Reynolds III CF, Silverman MM (2003). Confronting depression and suicide in physicians: a consensus statement. JAMA..

[48] Reed VA, Jernstedt GC, Reber ES (2001). Understanding and improving medical student specialty choice: a synthesis of the literature using decision theory as a referent. Teach Learn Med.

[49] Mortlock AM, Puzzo I, Taylor S, Kumari V, Young S, Sengupta S, Das M (2017). Enrichment activities in the medical school psychiatry programme - could this be a key to engaging medical students in psychiatry? A study from a high secure forensic psychiatric UK hospital. BMC Psychiatry.

[50] Cooter R, Erdmann JB, Gonnella JS, Callahan CA, Hojat M, Xu G (2004). Economic diversity in medical education: the relationship between students' family income and academic performance, career choice, and student debt. Eval Health Prof.

[51] National Bureau of Statistics of China (2020). China Statistical Yearbook.

[52] Goldacre MJ, Turner G, Fazel S, Lambert T (2005). Career choices for psychiatry: national surveys of graduates of 1974-2000 from UK medical schools. Br J Psychiatry.

[53] Patel V, Xiao S, Chen H, Hanna F, Jotheeswaran AT, Luo D, Parikh R, Sharma E, Usmani S, Yu Y, Druss BG, Saxena S (2016). The magnitude of and health system responses to the mental health treatment gap in adults in India and China. Lancet..

