# Supplementary material for: Career choice and influential factors among medical students majoring in psychiatry in China
Source: BMC Med Educ. 2021 Mar 25;21:183. doi: 10.1186/s12909-021-02622-x (PMC7992123; doi:10.1186/s12909-021-02622-x)
Supplement: Supplementary file 1 — Additional file 1. [file 12909_2021_2622_MOESM1_ESM.docx]

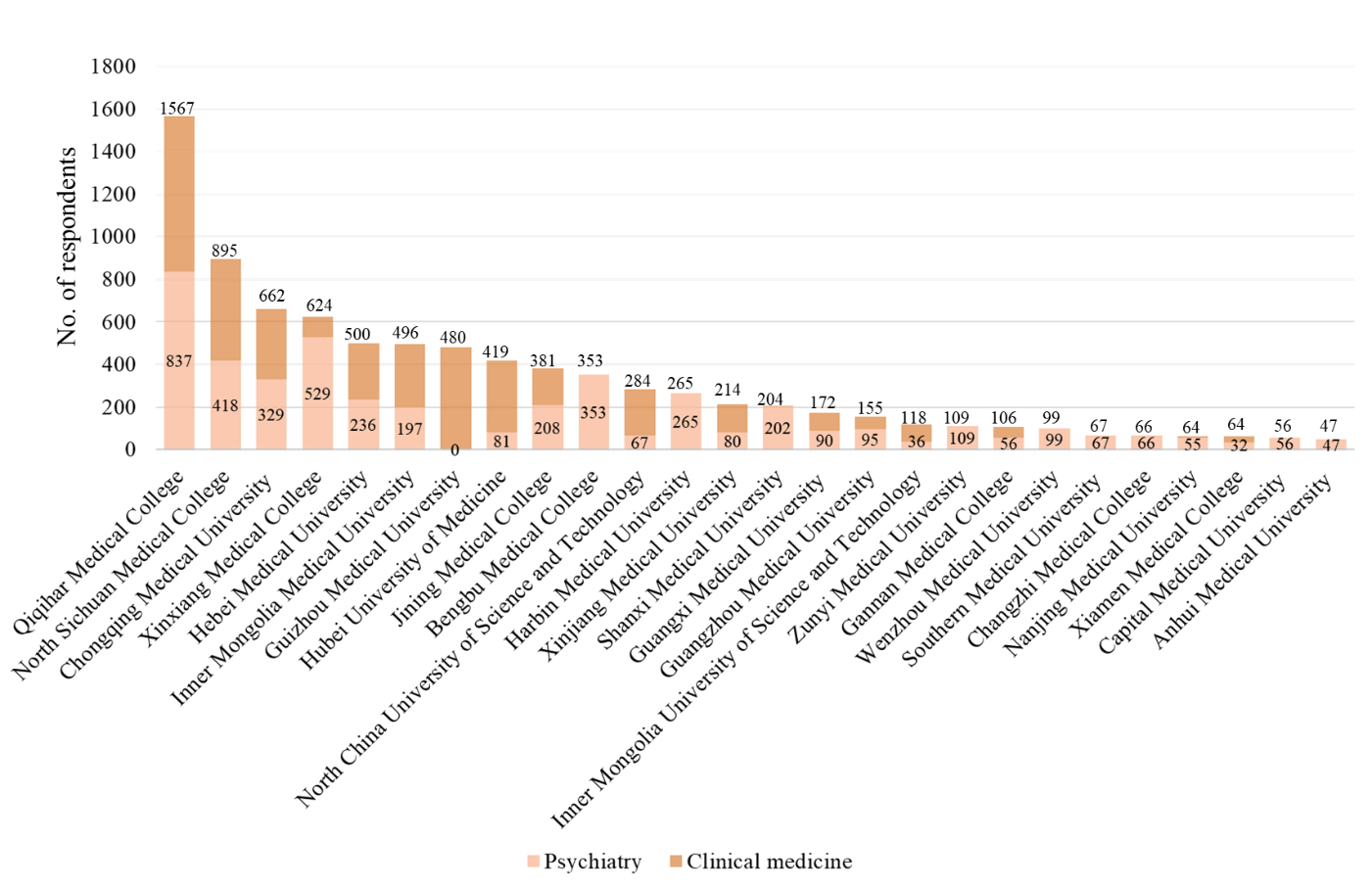


| **Medical school** | **Total No.** | **Psychiatry, n (%)** | **Clinical medicine, n (%)** |
| --- | --- | --- | --- |
| **Qiqihar Medical College** | **1567** | **837 (53.41)** | **730 (46.59)** |
| **North Sichuan Medical College** | **895** | **418 (46.70)** | **477 (53.30)** |
| **Chongqing Medical University** | **662** | **329 (49.70)** | **333 (50.30)** |
| **Xinxiang Medical College** | **624** | **529 (84.78)** | **95 (15.22)** |
| **Hebei Medical University** | **500** | **236 (47.20)** | **264 (52.80)** |
| **Inner Mongolia Medical University** | **496** | **197 (39.72)** | **299 (60.28)** |
| **Guizhou Medical University** | **480** | **0 (0)** | **480 (100)** |
| **Hubei University of Medicine** | **419** | **81 (19.33)** | **338 (80.67)** |
| **Jining Medical College** | **381** | **208 (54.59)** | **173 (45.41)** |
| **Bengbu Medical College** | **353** | **353 (100)** | **0(0)** |
| **North China University of Science and Technology** | **284** | **67 (23.59)** | **217 (76.41)** |
| **Harbin Medical University** | **265** | **265 (100)** | **0(0)** |
| **Xinjiang Medical University** | **214** | **80 (37.38)** | **134 (62.62)** |
| **Shanxi Medical University** | **204** | **202 (99.02)** | **2 (0.98)** |
| **Guangxi Medical University** | **172** | **90 (52.33)** | **82 (47.67)** |
| **Guangzhou Medical University** | **155** | **95 (61.29)** | **60 (38.71)** |
| **Inner Mongolia University of Science and Technology** | **118** | **36 (30.51)** | **82 (69.49)** |
| **Zunyi Medical University** | **109** | **109 (100)** | **0(0)** |
| **Gannan Medical College** | **106** | **56 (52.83)** | **50 (47.17)** |
| **Wenzhou Medical University** | **99** | **99 (100)** | **0(0)** |
| **Southern Medical University** | **67** | **67 (100)** | **0(0)** |
| **Changzhi Medical College** | **66** | **66 (100)** | **0(0)** |
| **Nanjing Medical University** | **64** | **55 (85.94)** | **9 (14.06)** |
| **Xiamen Medical College** | **64** | **32 (50.00)** | **32 (50.00)** |
| **Capital Medical University** | **56** | **56 (100)** | **0(0)** |
| **Anhui Medical University** | **47** | **47 (100)** | **0(0)** |
| **Total No.** | **8467** | **4610** | **3857** |

**Online Resource 1. No. of respondents of each medical school.**

**Online Resource 2. Examples of coding and statements about psychiatry.**

|  | **Choosing psychiatry** | **Rejecting psychiatry** |
| --- | --- | --- |
| **Personal interest** | “I’m interested in psychiatry.”  “I’m interested in psychology and I also want to be a doctor.” | “I'm not interested in psychiatry.”  “I'm interested in psychiatry but more in clinical medicine.” |
| **Family’s suggestion or influence** | “I have a family member working in psychiatry.”  “My parents advised me to choose a psychiatry major.” | “Despite my interest in psychiatry, my family and friends objected.”  “Family member did the choice for me.” |
| **Experience of mental problems of oneself or others** | “My mother had emotional problems and insomnia. I want to learn more about mental disorders and help her.”  “I once had a mental problem and I want to heal myself, know more about myself.” | — |
| **Low admission score of psychiatry** | “The admission score of psychiatry is lower than clinical medicine. My score meets the requirement of psychiatry but not for clinical medicine, and I want to be a doctor.”  “My application for other specialties was rejected so I was transferred to psychiatry, or I will miss the opportunity of medical school. ” | “I got a much higher score than psychiatry at matriculation.”  “Psychiatry has a lower admission score rank.” |
| **Lack knowledge of psychiatry** | “I didn’t know this subject. I just filled in the application form casually.”  “I don’t know. It was an accident for me to be enrolled in this specialty.” | “I have no idea what this major is.”  “I don't know this major at all.” |
| **Positive/negative attitudes toward psychiatry** | “Psychiatrists are in great demand. The prospect of psychiatry is promising.”  “China has a shortage of psychiatrists. It’s easy for psychiatry major students to find a job.” | “Income and social status are bad in psychiatry.”  “Psychiatry is so obscure to learn. I’m afraid of psychiatric patients.” |
| **School** | “Psychiatry is the best major in school.” | “Despite my interest in psychiatry, my school didn't encourage me to apply for this specialty.” |
|  | “The school is close to my home.” |  |
| **Other reasons** | “I don’t want to say.” | “No clear reasons.” |

**Online Resource 3. When thinking about your career specialty after medical school, how important are the following considerations?**

|  | 1. Not important | 2. Somewhat important | 3. Partially important | 4. Very important |
| --- | --- | --- | --- | --- |
| Working for social change |  |  |  |  |
| High income potential |  |  |  |  |
| Social recognition or status |  |  |  |  |
| Stable, secure future |  |  |  |  |
| Creativity and initiative |  |  |  |  |
| Expression of personal values |  |  |  |  |
| Availability of jobs |  |  |  |  |
| Leadership potential |  |  |  |  |
| Opportunity for innovation |  |  |  |  |
| Competitiveness of specialty |  |  |  |  |
| Level of educational debt |  |  |  |  |
| Role model influence |  |  |  |  |
| Income expectations |  |  |  |  |
| Length of residency training |  |  |  |  |
| Family expectations |  |  |  |  |
| My future family plans |  |  |  |  |
| Work/life balance |  |  |  |  |
| Fit with personality, interests, and skills |  |  |  |  |
| Content of specialty |  |  |  |  |

**Online Resource 4. Initial 36 variables, in order of *p* value, from the bivariate analysis.**

| Female* |
| --- |
| Higher grade* |
| Psychiatry clerkship* |
| Matriculation reason: Personal interest* |
| Matriculation reason: Experience of mental problems* |
| Matriculation reason: Low admission score* |
| Matriculation reason: Lack knowledge of psychiatry * |
| Leadership potential* |
| Level of educational debt* |
| Matriculation reason: Family’s suggestion or influence* |
| My future family plans* |
| ATP score* |
| Stigma score* |
| Length of residency training* |
| Social recognition or status* |
| Role model influence* |
| Fit with personality, interests, and skills* |
| Content of specialty* |
| Income expectations* |
| Opportunity for innovation* |
| Work-life balance* |
| Competitiveness of specialty |
| History of psychiatry visit |
| Experience of illegal acts related to medical staff |
| Family history of mental disorders |
| Family lives in urban area |
| Higher family income |
| High income potential |
| Creativity and initiative |
| Availability of jobs |
| Working for social change |
| Matriculation reason: School |
| Family expectations |
| Stable, secure future |
| Expression of personal values |
| Better academic achievement |

**p* < 0.05.

**Online Resource 5. Liner correlation between psychiatry career choice and economic level and mental health service capacity of each province.**

|  | Regional GDP per capita | | Rate of career choice for psychiatry | |
| --- | --- | --- | --- | --- |
|  | *r* | *p* | *r* | *p* |
| Regional GDP per capita | — | — | 0.13 | 0.605 |
| No. of health workers | 0.79 | < 0.001 | 0.43 | 0.075 |
| No. of psychiatrists | 0.91 | < 0.001 | 0.32 | 0.207 |
| No. of psychiatry beds | 0.35 | 0.159 | 0.02 | 0.934 |

GDP: Gross domestic product

No.: number

**Online Resource 6. Hierarchical analysis of influence of demographic factors on psychiatry specialty choice at matriculation classified by grade.**

|  | | **Freshman** | | **Low grade** | | **High grade** | |
| --- | --- | --- | --- | --- | --- | --- | --- |
|  | | **OR (95% CI)** | ***p*** | **OR (95% CI)** | ***p*** | **OR (95% CI)** | ***p*** |
| **Gender** | |  |  |  |  |  |  |
|  | Male | reference |  | reference |  | reference |  |
|  | Female | 1.39 (1.18-1.64) | < 0.001 | 1.51 (1.34-1.69) | < 0.001 | 1.26 (0.87-1.82) | 0.23 |
| **Family living area** | |  |  |  |  |  |  |
|  | Village | reference |  | reference |  | reference |  |
|  | Town | 1.15 (0.92-1.46) | 0.22 | 0.83 (0.71-0.97) | 0.02 | 1.76 (1.03-3.01) | 0.04 |
|  | City | 1.59 (1.29-1.96) | < 0.001 | 1.02 (0.88-1.18) | 0.84 | 1.57 (1.02-2.42) | 0.04 |
| **Family income (RMB/month)** | |  |  |  |  |  |  |
|  | < 1000 | reference |  | reference |  | reference |  |
|  | 1000-2999 | 1.94 (1.22-3.07) | 0.01 | 1.05 (0.79-1.40) | 0.73 | 0.76 (0.3-1.93) | 0.57 |
|  | 3000-4999 | 1.93 (1.22-3.05) | < 0.001 | 1.18 (0.89-1.55) | 0.25 | 0.69 (0.28-1.71) | 0.42 |
|  | 5000-9999 | 2.39 (1.51-3.78) | < 0.001 | 1.24 (0.94-1.64) | 0.13 | 0.68 (0.27-1.7) | 0.41 |
|  | 10000-14999 | 1.82 (1.10-3.02) | 0.02 | 1.43 (1.05-1.95) | 0.02 | 1.24 (0.41-3.8) | 0.71 |
|  | 15000-20000 | 1.92 (1.08-3.40) | 0.03 | 1.51 (1.02-2.24) | 0.04 | 0.63 (0.18-2.24) | 0.47 |
|  | > 20000 | 2.50 (1.33-4.71) | < 0.001 | 1.77 (1.21-2.58) | < 0.001 | 0.61 (0.17-2.21) | 0.45 |
| **History of using psychiatry service** | |  |  |  |  |  |  |
|  | No | reference |  | reference |  | reference |  |
|  | Yes | 3.07 (1.48-6.36) | < 0.001 | 1.21 (0.80-1.82) | 0.36 | 1.64 (0.8-3.35) | 0.18 |
| **Family history of mental disorders** | |  |  |  |  |  |  |
|  | No | ref |  | ref |  | ref |  |
|  | Yes | 1.23 (0.85-1.76) | 0.27 | 1.42 (1.16-1.75) | < 0.001 | 4.22 (0.55-32.63) | 0.17 |
